# Imaging-Based Diagnosis of a Right Coronary Artery-to-Left Anterior Descending Artery Fistula Associated With a Double Right Coronary Artery Presenting With Angina

**DOI:** 10.7759/cureus.109317

**Published:** 2026-05-20

**Authors:** Fernanda P Soto Diaz, Tatiana A Guzman Del Rio, Alexis D Lastra Bermudez, Janet De La Torre, Imtiaz Ahmed

**Affiliations:** 1 Research, Universidad Autonoma de Guadalajara School of Medicine, Guadalajara, MEX; 2 Research and Development, Universidad Autonoma de Guadalajara, Guadalajara, MEX; 3 Radiology, HonorHealth Tempe Medical Center, Tempe, USA

**Keywords:** coronary arterial fistula, double right coronary artery, imaging findings, left anterior descending coronary artery to pulmonary artery fistula, right coronary artery (rca) anomaly

## Abstract

Coronary artery fistulas (CAFs) are uncommon coronary anomalies that are often asymptomatic but may present later in life due to the coronary steal phenomenon, leading to myocardial ischemia and related complications. We report the case of a 76-year-old male presenting with anginal chest pain. Coronary computed tomography angiography performed after inconclusive prior cardiac testing revealed a direct fistulous communication between the right coronary artery (RCA) and the left anterior descending artery (LAD), an exceptionally rare configuration. Imaging also demonstrated a double RCA, an uncommon congenital variant characterized by two parallel right coronary arteries arising from a single or separate ostia, which may coexist with other coronary anomalies and complicate interpretation. The study showed a tortuous fistulous tract with features consistent with chronic high-flow physiology and coronary steal, correlating with the patient’s clinical presentation. This case highlights the importance of considering rare coronary anomalies in elderly patients with new-onset angina and underscores the value of coronary computed tomography angiography in providing detailed three-dimensional visualization of coronary anatomy, enabling definitive diagnosis and guiding clinical management in complex coronary vascular anomalies.

## Introduction

Coronary artery fistulas (CAFs) are rare coronary anomalies that may present with myocardial ischemia due to the coronary steal phenomenon [1,2]. Coronary artery anomalies overall are uncommon, affecting approximately 0.2-1.4% of the population [3]. CAFs are identified in approximately 0.2-0.6% of coronary angiographic studies [4,5]. The coexistence of a double right coronary artery (RCA) with a CAF is extremely rare, particularly when involving a direct coronary-to-coronary communication between the RCA and the left anterior descending (LAD) artery. With advancing age, progressive dilation of the fistula may increase shunt volume and result in symptomatic ischemia [3,6]. In this case, a double RCA was also identified, making this presentation exceptionally rare, with a reported prevalence of 0.01-0.46% [7].

The prevalence and presentation of these two anomalies together make it difficult to calculate a valid co-occurrence rate because the prevalence percentages may not be independent. There is a possibility of overlapping embryologic developmental pathways, as these structures share a common embryologic origin. After reviewing the relevant literature regarding these vascular anomalies, this case may suggest the presence of a shared developmental abnormality affecting the coronary vasculature. It has been hypothesized that the existing fistula may be secondary to a persistent interpolated coronary anastomosis that failed to regress during embryologic development. Anomalies such as these are usually identified as isolated findings; however, they may coexist with other vascular abnormalities, including duplicated coronary arteries.

Partial shunting of blood appears to be the underlying mechanism in this case, resulting in the coronary steal phenomenon. This occurs when the blood flow responsible for meeting myocardial oxygen demand is diverted away from the myocardial capillary bed into a low-resistance tract, thereby reducing oxygen delivery to the tissue. It is possible that altered perfusion patterns or competitive flow dynamics among the coronary arteries contributed to impaired myocardial oxygen supply, resulting in myocardial ischemia presenting as angina pectoris. These anomalies are particularly rare, with most reported cases identified incidentally and without symptomatic presentation. In this patient, the coexistence of these vascular abnormalities may have contributed to the symptomatic clinical presentation.

## Case presentation

We present a case of a double RCA associated with a direct RCA-to-LAD fistula, diagnosed using coronary computed tomography angiography (CTA) in a 76-year-old male patient presenting with angina pectoris despite the absence of obstructive coronary artery disease. The patient presented to the emergency department with exertional chest pain and symptoms suggestive of myocardial ischemia. Prior to presentation to the radiology department, the patient had undergone noninvasive cardiac testing to exclude obstructive coronary artery disease. Limited clinical information, including past medical history, family history, social history, and prior cardiac testing, was available at the time of CTA evaluation, reflecting a real-world radiology workflow scenario at the time of diagnosis.

Given the patient’s persistent symptoms and ongoing concern for myocardial ischemia, coronary CTA was performed for further evaluation of the coronary anatomy. The clinical presentation and symptomatology were sufficient to support CTA evaluation. Imaging demonstrated a rare coronary-to-CAF connecting the RCA to the LAD. In addition, imaging revealed a duplicated origin of the RCA, with one branch continuing as the dominant vessel supplying the myocardium and a second branch forming the fistulous communication with the LAD. These findings were consistent with a CAF demonstrating chronic high-flow shunting physiology, with features suggestive of the coronary steal phenomenon secondary to the RCA-to-LAD fistula.

Imaging findings

CT Angiography Technique

Electrocardiogram-gated coronary CTA was performed following intravenous administration of iodinated contrast material. Multiplanar reformatted images and three-dimensional volume-rendered reconstructions were generated for a comprehensive assessment of the coronary anatomy.

Axial CTA Findings

Axial contrast-enhanced coronary CTA images (Figure 1A-1B) demonstrate a duplicated origin of the RCA at the coronary ostium, with two distinct vessels arising from the proximal RCA. One branch continues as the dominant RCA supplying the myocardium along its expected course within the right atrioventricular groove.

**Figure 1:**
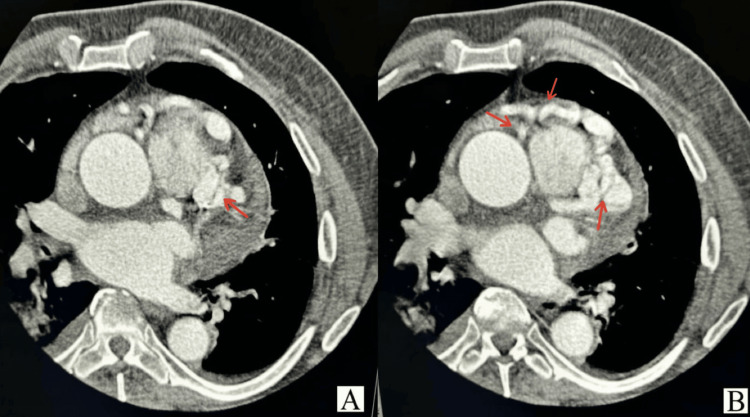
Axial contrast-enhanced coronary CTA images (A, B) demonstrating duplicated origin of the right coronary artery at the coronary ostium One branch continues as the dominant right coronary artery supplying the myocardium, while a second branch forms a dilated tortuous fistulous vessel communicating with the left anterior descending artery (arrows). These findings are consistent with a coronary-to-coronary artery fistula. CTA: computed tomography angiography

The second branch forms a tortuous vascular channel that courses anteriorly toward the LAD, creating a direct coronary-to-coronary fistulous communication between the RCA and the LAD.

The fistulous vessel appears dilated and serpiginous, consistent with chronic high-flow physiology. The LAD distal to the site of communication appears relatively decreased in caliber, suggesting reduced distal perfusion pressure consistent with coronary steal physiology.

No significant obstructive atherosclerotic disease is identified within the major epicardial coronary arteries, and the cardiac chambers demonstrate no acute enlargement.

Three-Dimensional Volume-Rendered CTA Findings

Three-dimensional volume-rendered reconstructions clearly delineate the duplicated origin of the RCA and the spatial relationship of the fistulous tract (Figure 2A-2B).

**Figure 2:**
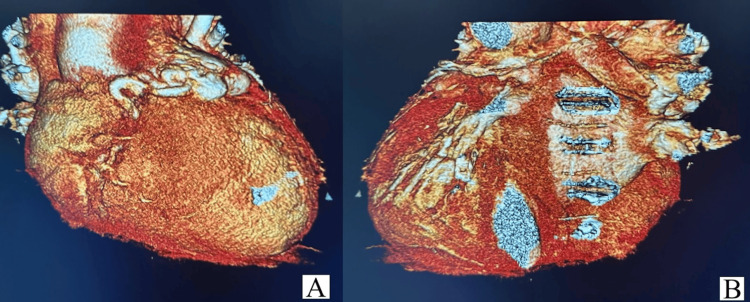
Three-dimensional volume-rendered coronary CTA images (A, B) illustrating the duplicated origin of the right coronary artery and the fistulous communication between the RCA and the LAD. The fistulous tract demonstrates a serpiginous course with dilation consistent with chronic high-flow physiology. CTA provides detailed visualization of the fistula’s origin, course, and termination. CTA: computed tomography angiography, RCA: right coronary artery, LAD: left anterior descending artery

One branch follows the expected course of the dominant RCA supplying the myocardium, while the second branch forms a prominent tortuous fistulous tract communicating directly with the LAD.

The involved coronary segments demonstrate ectasia with focal aneurysmal dilation, consistent with long-standing high-flow shunting. The three-dimensional reconstructions allow clear visualization of the origin, course, and termination of the fistula, confirming a direct RCA-to-LAD CAF.

Imaging impression

Findings are consistent with a direct CAF between the RCA and the LAD, associated with dilation and tortuosity of the feeding vessel and imaging features suggestive of the coronary steal phenomenon. No significant obstructive coronary artery disease was identified.

## Discussion

The primary mechanism underlying symptoms in patients with CAFs is the coronary steal phenomenon. Because the fistulous tract represents a low-resistance pathway, blood is preferentially diverted away from the distal myocardial capillary bed. This results in reduced perfusion pressure and flow, causing distal hypoperfusion and myocardial ischemia presenting as angina pectoris, even in the absence of obstructive coronary artery disease.

Direct coronary-to-coronary fistulas are particularly uncommon, as most CAFs drain into low-pressure cardiac chambers or the pulmonary artery.

Although most CAFs are congenital, symptoms frequently manifest later in life. Chronic high-pressure flow through the fistula can lead to progressive dilation and ectasia of the feeding artery over several decades. As shunt volume increases, myocardial ischemia may develop, ultimately presenting as angina pectoris or heart failure. Elderly patients are particularly susceptible because of age-related reductions in coronary reserve and myocardial compliance.

In this case, the patient was also found to have a double RCA, a rare congenital variant characterized by two parallel right coronary arteries arising from either a single ostium or separate ostia. Recognition of this anomaly is important because, although rare, it may coexist with coronary abnormalities such as CAFs and may complicate the interpretation of coronary imaging and the patient’s clinical symptomatology.

Coronary CTA plays a pivotal role in the evaluation of suspected CAFs by providing high-resolution three-dimensional visualization of the coronary anatomy. CTA allows precise delineation of the fistula’s origin, course, and termination while simultaneously identifying associated anomalies, such as a duplicated RCA, thereby facilitating accurate diagnosis and guiding future clinical management.

Hemodynamically significant fistulas may lead to complications, including arrhythmias, heart failure, aneurysmal dilation, thrombus formation, infective endocarditis, myocardial infarction, and sudden cardiac death. These risks increase with age and fistula size, underscoring the importance of early recognition, appropriate surveillance, and timely intervention.

## Conclusions

This case highlights a rare direct fistulous connection between the RCA and the LAD, as well as the coexistence of an uncommon double RCA. These anomalies resulted in exertional angina pectoris in this elderly patient. This rare congenital coronary anomaly is characterized by two parallel right coronary arteries arising from either a single ostium or separate ostia, along with an abnormal communication between coronary arteries. Although these anomalies are often clinically silent, their recognition is important because they may coexist with other congenital abnormalities. In this case, the coexistence of these pathologies likely contributed to the patient’s symptomatic presentation.

Progressive enlargement of the fistulous tract may have led to the coronary steal phenomenon, resulting in clinically significant myocardial ischemia despite the absence of obstructive coronary artery disease. Coronary CTA played a critical role in the diagnosis by providing a detailed three-dimensional visualization of the coronary anatomy and allowing accurate delineation of both the fistula and the duplicated RCA. This facilitated appropriate clinical management by the cardiology team and may help prevent potential long-term complications in this patient.
